# Supplementary material for: Slc4a7 Regulates Retina Development in Zebrafish
Source: Int J Mol Sci. 2024 Sep 5;25(17):9613. doi: 10.3390/ijms25179613 (PMC11403715; doi:10.3390/ijms25179613)
Supplement: Supplementary file 1 [file ijms-25-09613-s001.zip › ijms-3133819-supplementary.pdf]

Supplementary Materials

Supplementary Table S1. Primer sequences for real-time PCR.

Primer Name	Sequence (5′-3′)
slc4a7-F-1	CCACAGGCAATGATGATGA
slc4a7-R-1	TTCCGATGGTGCTTATGAC
β-actin-F	TGCTGTTTTCCCCTCCATTG
β-actin-R	GTCCCATGCCAACCATCACT

Supplementary Table S2. Primer sequences for real-time quantitative PCR.

Primer Name	Sequence (5′-3′)
slc4a7-F-2	TGCTTGAGGTTAGACCAGCG
slc4a7-R-2	ACATGAACCGGACTGAACCC
β-actin-F	TGCTGTTTTCCCCTCCATTG
β-actin-R	GTCCCATGCCAACCATCACT

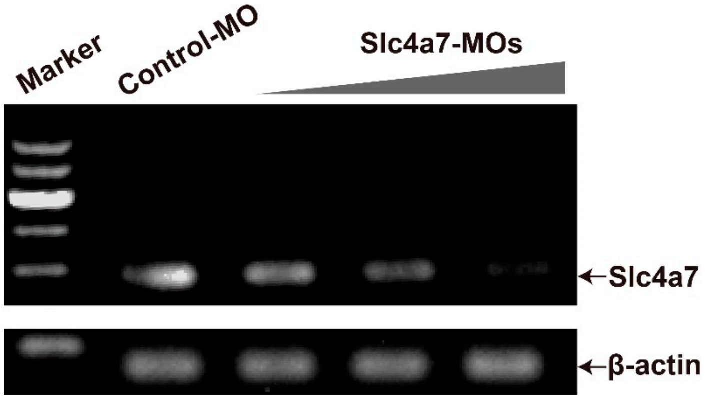

Supplementary Figure S1. RT-PCR confirmed expression change in *slc4a7*-MO injected zebrafish larvae. Three doses (0.50 ng, 0.75 ng, 1.00 ng) of *slc4a7*-MOs were tested at 5 dpf.

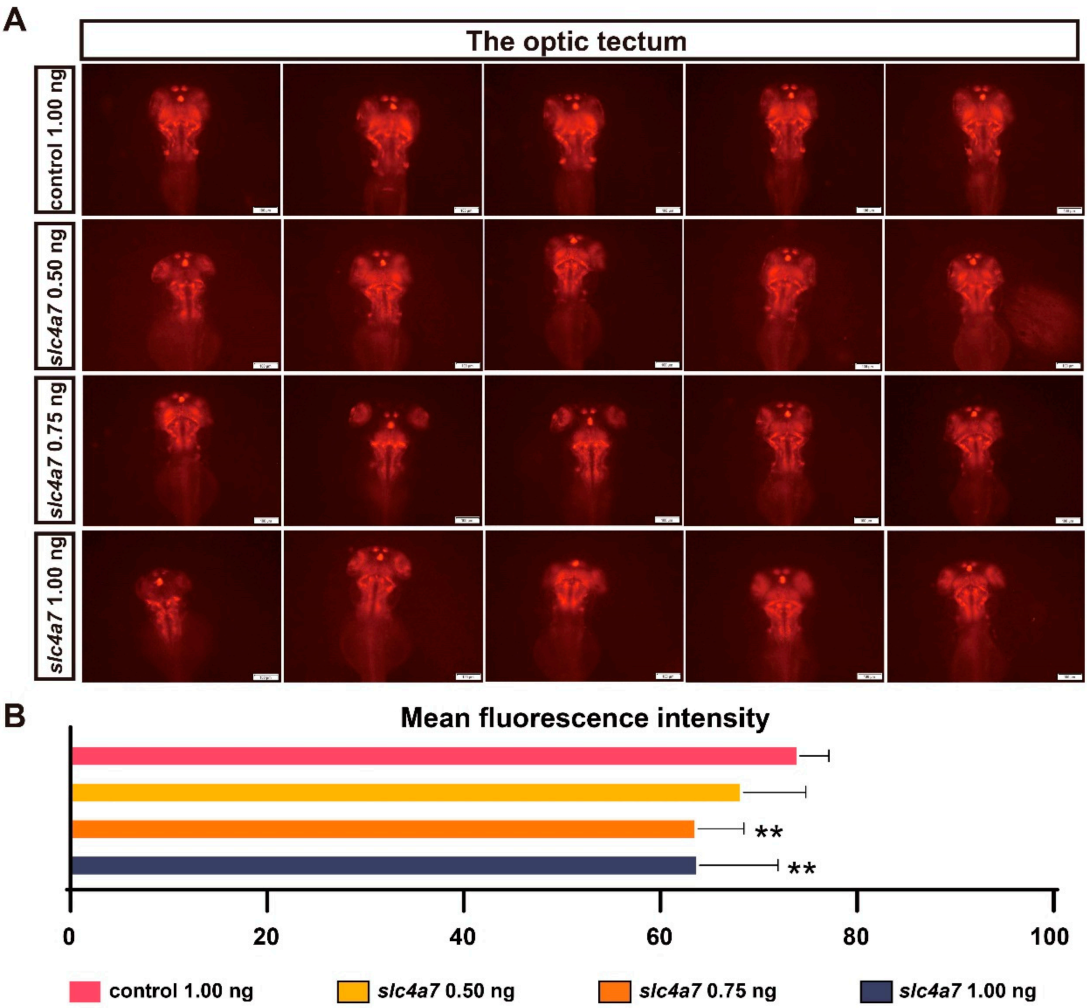

Supplementary Figure S2. Silencing the expression of *slc4a7* leads to abnormalities in the optic tectum. (**A**) Top-down perspective of the optic tectum in Tg(gad1b:mCherry) transgenic larvae subjected to injections of *slc4a7* MO and control MO at 5 dpf. (**B**) Mean fluorescence intensity quantification in the optic tectum region. Bar plots were shown as the mean ± s.e.m. Data were analyzed using one-way ANOVA with Tukey's post hoc tests, ** $p < 0.001$.

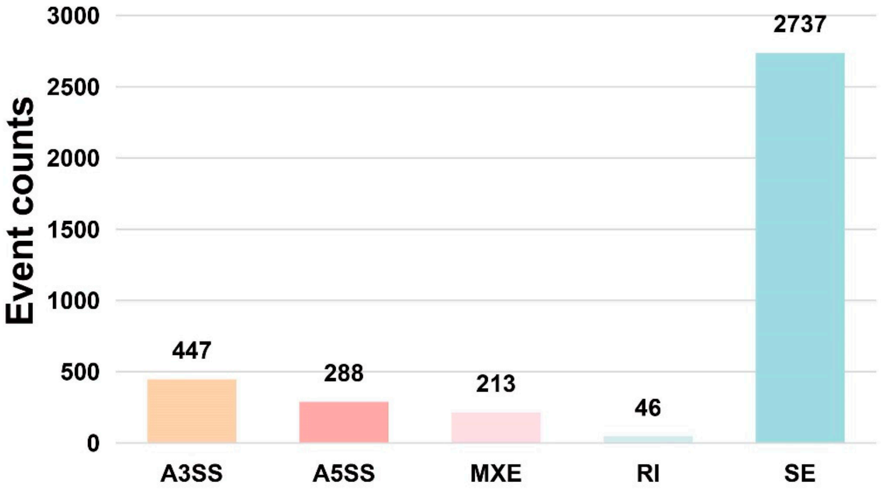

Supplementary Figure S3. Differential Alternative Splicing Analysis. Overview of the results from the analysis of differential alternative splicing (DAS) events, including Exon Skipping (SE), Alternative 3' Splicing Junction (A3SS), Alternative 5' Splicing Junction (A5SS), Mutually Exclusive Exon (MXE), and Intron Retention (RI).

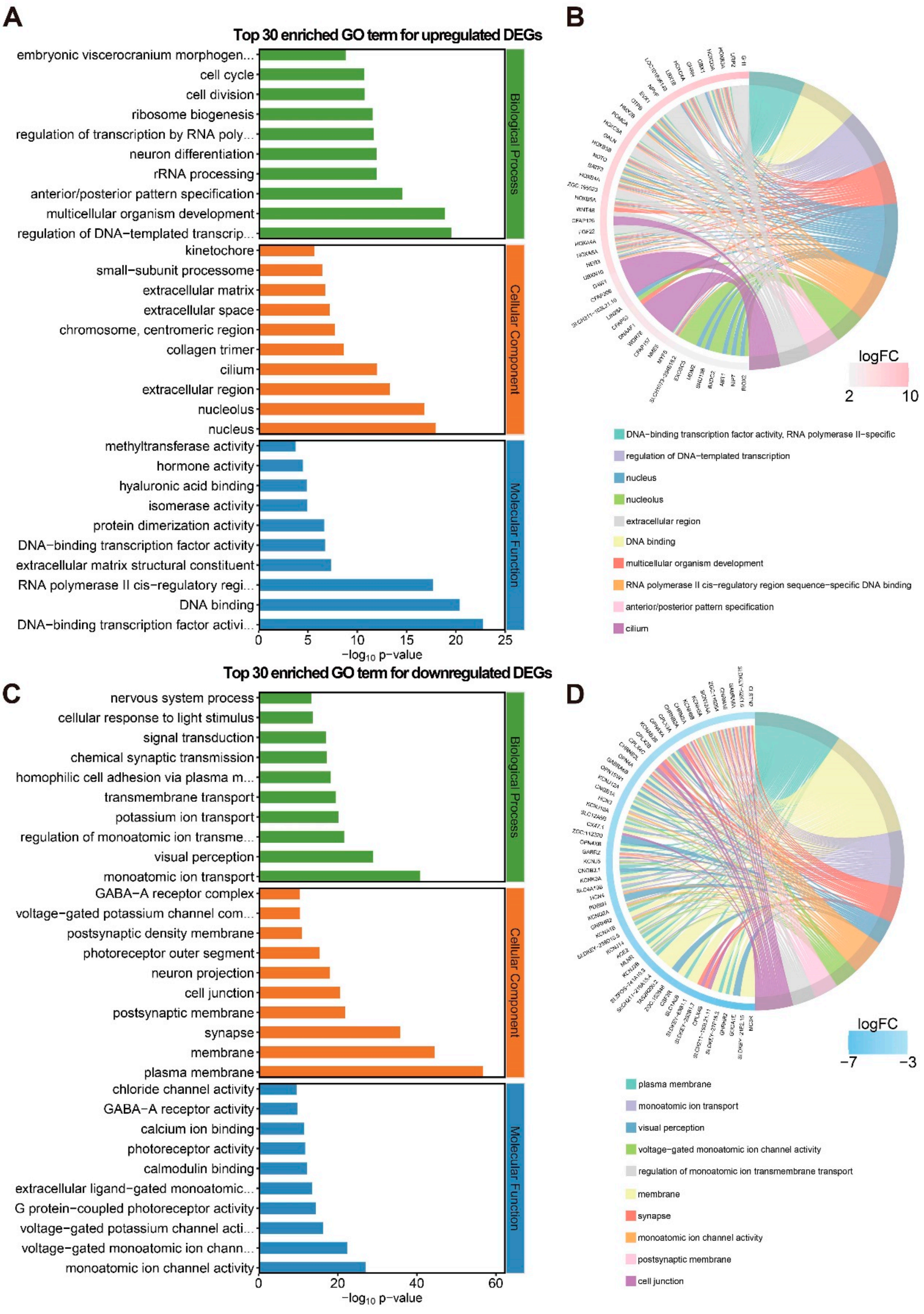

Supplementary Figure S4. GO enrichment analysis for key targets. (**A**) The top 30 enriched GO term for upregulated DEGs. (**B**) Chord diagram of upregulated DEGs. (**C**) The top 30 enriched GO term for downregulated DEGs. (**D**) Chord diagram of downregulated DEGs.

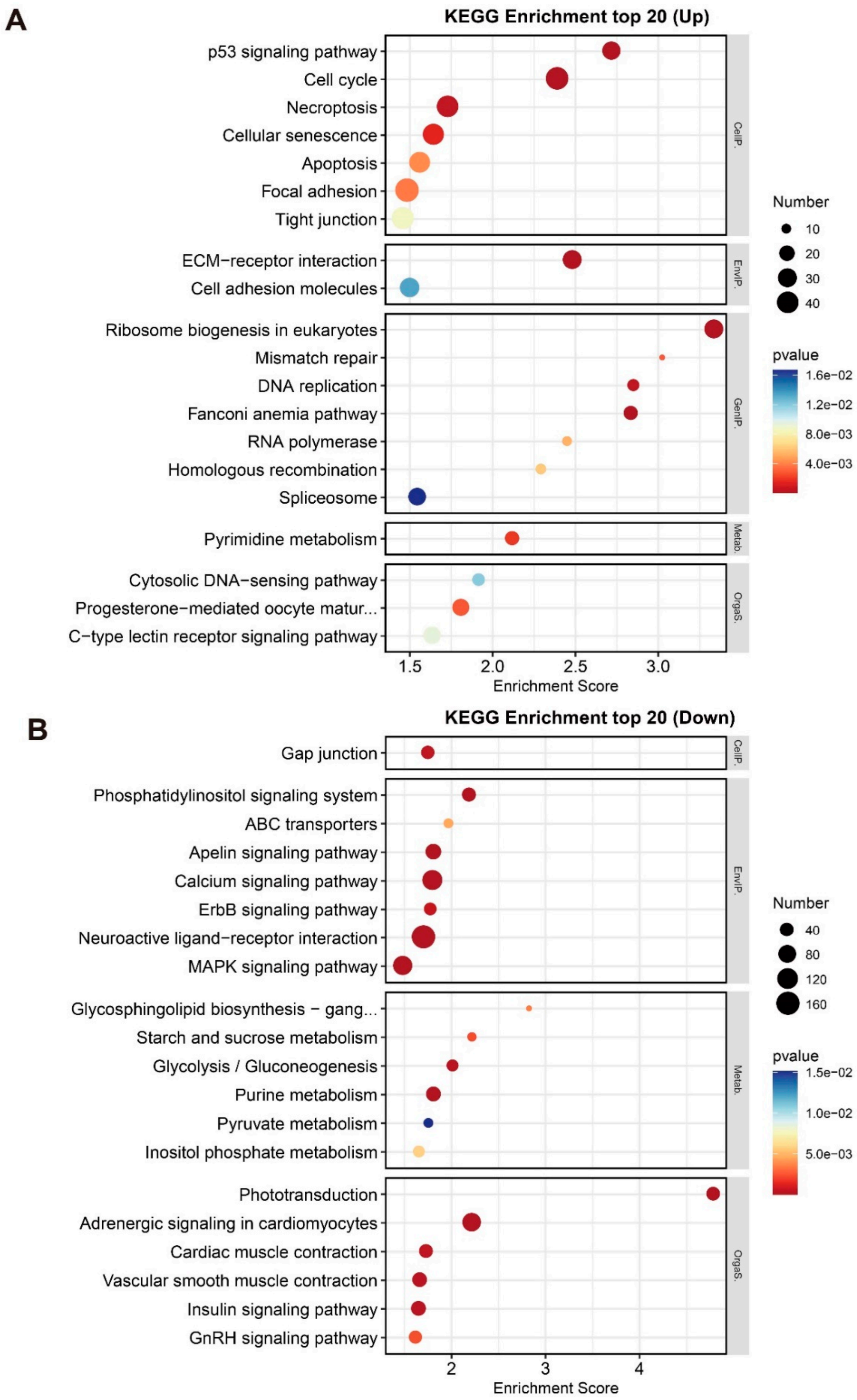

Supplementary Figure S5. KEGG Enrichment top 20. (**A**) The top 20 upregulated KEGG pathways of DEGs. (**B**) The top 20 downregulated KEGG pathways of DEGs.

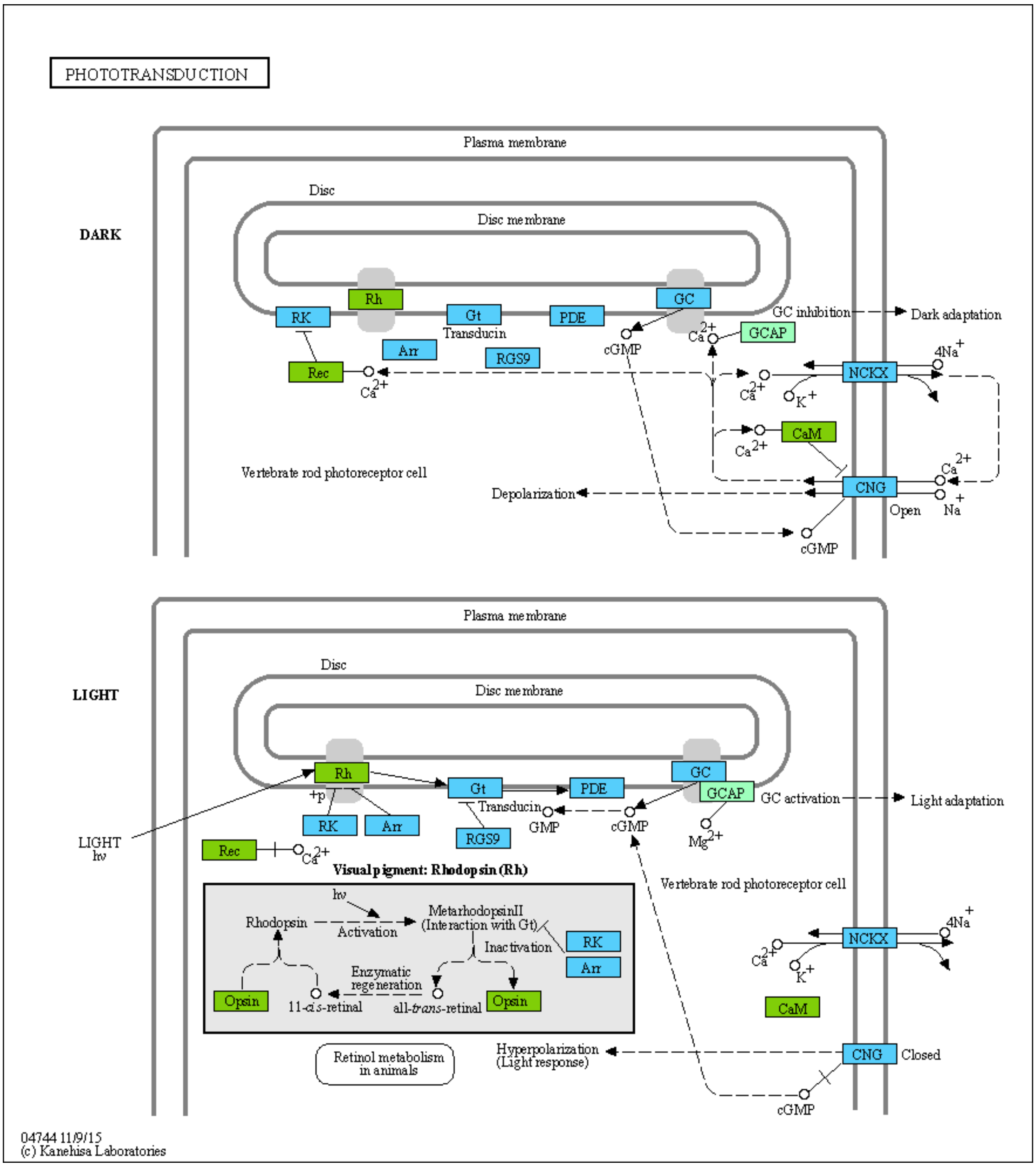

Supplementary Figure S6. KEGG pathway map depicts DEGs in phototransduction. The red box indicates upregulated DEGs in phototransduction, while the blue box denotes downregulated DEGs.

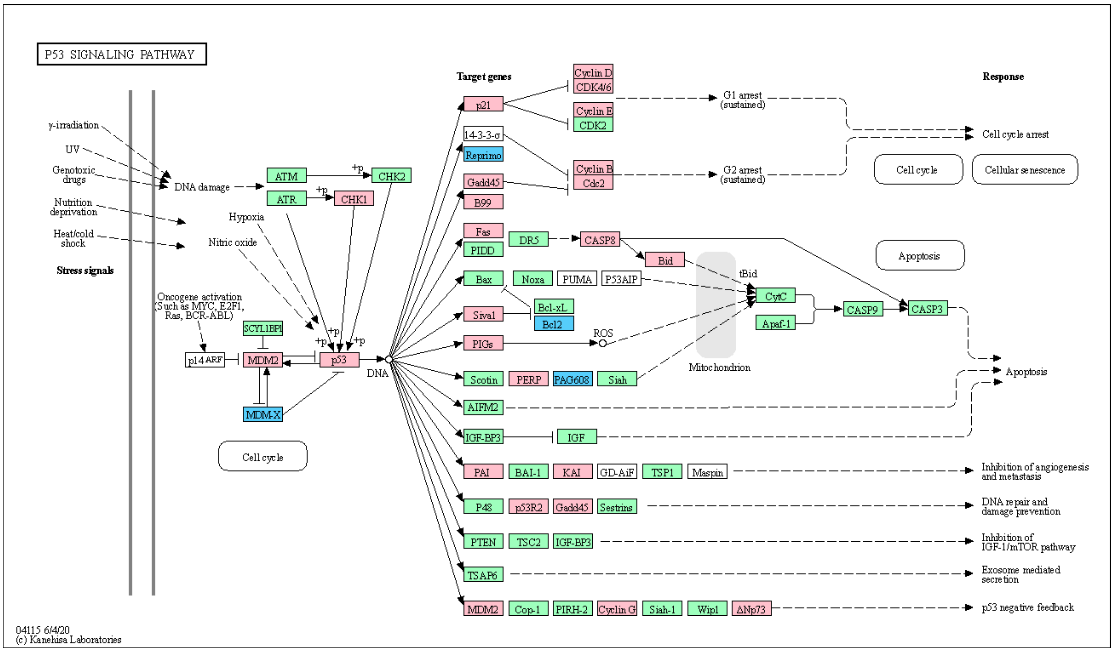

Supplementary Figure S7. KEGG pathway map depicts DEGs in p53 signaling pathway. The red box indicates upregulated DEGs in p53 signaling pathway, while the blue box denotes downregulated DEGs.

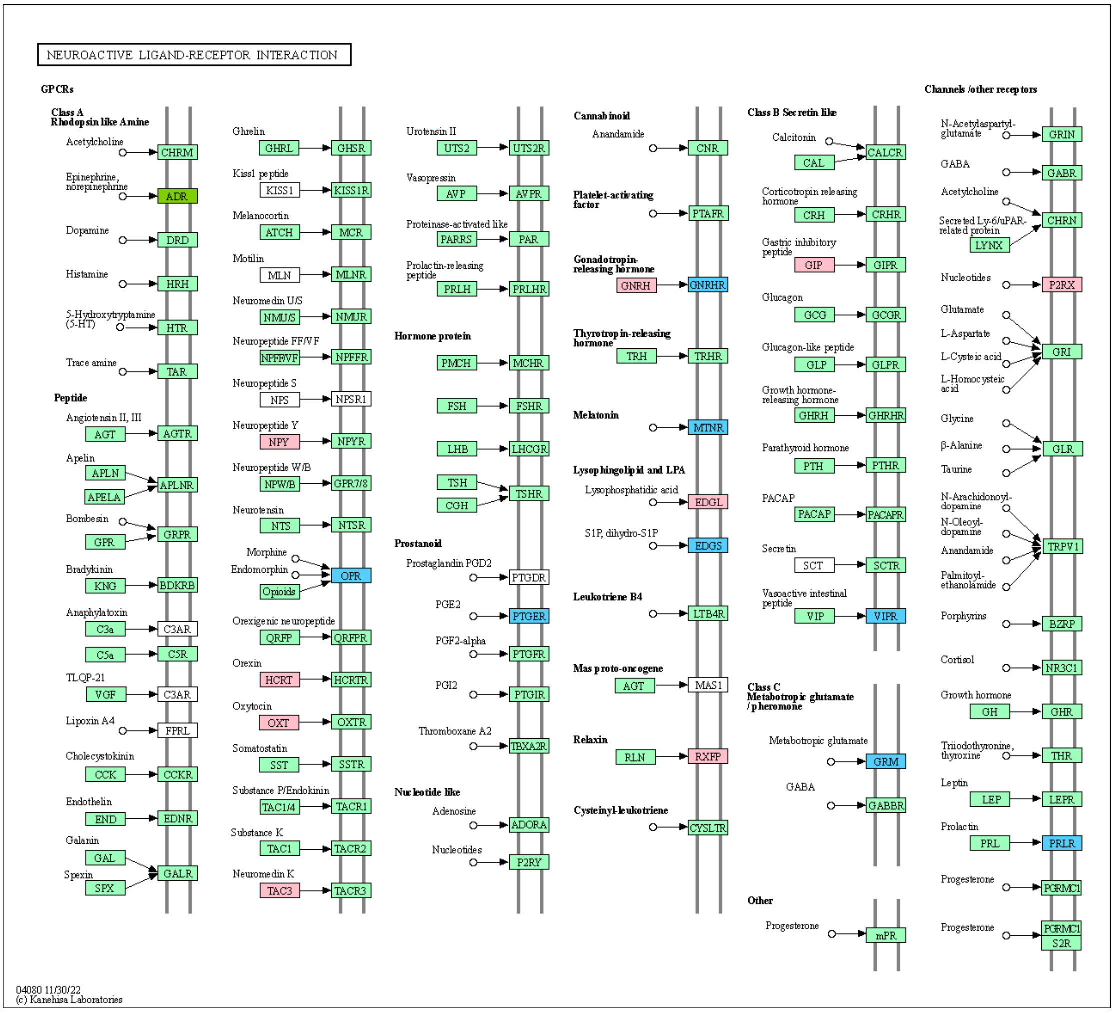

Supplementary Figure S8. KEGG pathway map depicts DEGs in neuroactive ligand–receptor interaction. The red box indicates upregulated DEGs in neuroactive ligand–receptor interaction, while the blue box denotes downregulated DEGs.

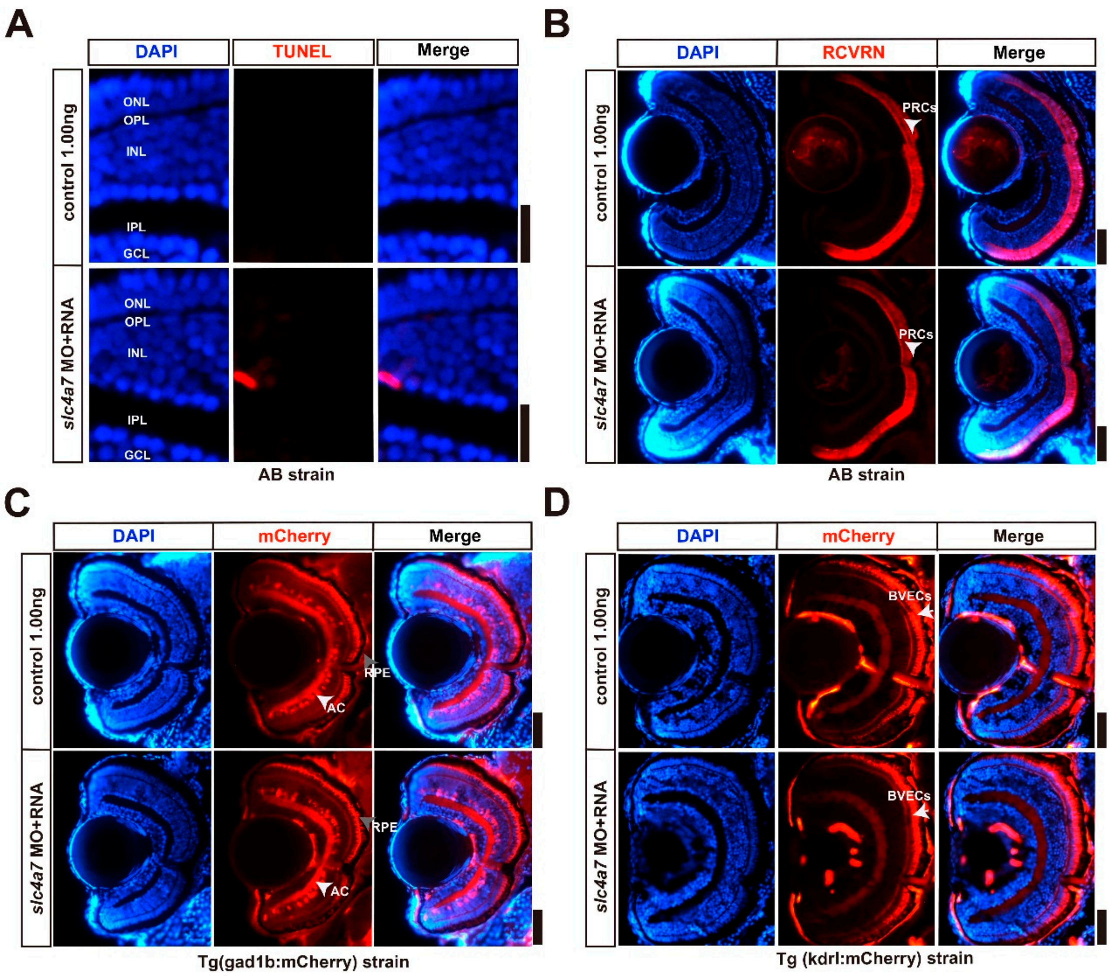

Supplementary Figure S9. Compensation with *slc4a7* mRNA rescues retinal phenotypes in *slc4a7*-deficient morphants. (**A**) TUNEL assay was used to detect apoptosis in larval retinas at 5 dpf. Scale bar = 20 μm. (**B**) Immunostaining for RCVRN in co-injected (mRNA and MO) and control AB zebrafish strains at 5 dpf. Scale bar = 50 μm. (**C**) The fluorescence imaging depicts retinal pigment epithelium cells and amacrine cells in co-injected (mRNA and MO) and control Tg (gad1b:mCherry) strains at 5 dpf. Scale bar = 50 μm. (**D**) The fluorescence imaging focusing on blood vessel endothelial cells in co-injected (mRNA and MO) and control Tg(kdrl:mCherry) strains at 5 dpf. Scale bar = 50 μm.

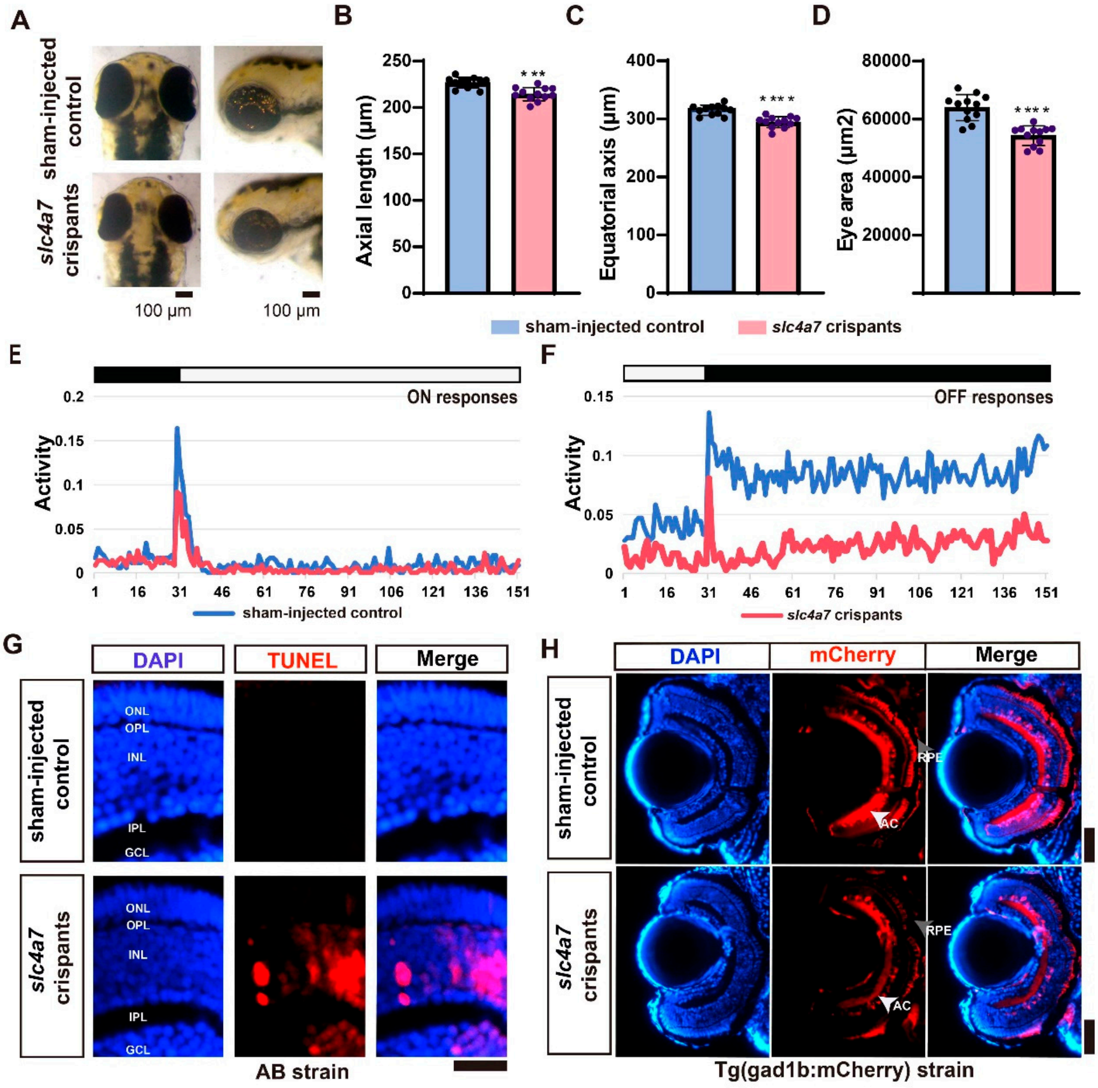

Supplementary Figure S10. The ocular phenotypes in the mosaic slc4a7 crispants. (**A**) Enlarged vertical and lateral views of larval eyeballs. Crispants and control larvae were subjected to eye parameter measurements at 3 dpf. Scale bar = 100 mm. (**B**–**D**) Statistical analysis was performed on the axial length and ocular area. (**E**,**F**) Line charts illustrate the results of VMR testing for slc4a7 crispants and control larvae at 5 dpf. (**G**) The TUNEL assay was employed to detect apoptosis in both crispants and control larvae at 5 dpf. Scale bar = 20 μm. (**H**) The fluorescence imaging depicting retinal pigment epithelium cells and amacrine cells in crispants and control larvae Tg (gad1b:mCherry) strains at 5 dpf. Scale bar represents 50 μm. Data were analyzed using Student's t-test, *** $p < 0.001$, **** $p < 0.0001$, indicating significant differences from the control group.
